# Acyloxy Nitroso Compounds Inhibit LIF Signaling in Endothelial Cells and Cardiac Myocytes: Evidence That STAT3 Signaling Is Redox-Sensitive

**DOI:** 10.1371/journal.pone.0043313

**Published:** 2012-08-15

**Authors:** Carlos Zgheib, Mazen Kurdi, Fouad A. Zouein, Barak W. Gunter, Brian A. Stanley, Joe Zgheib, Damian G. Romero, S. Bruce King, Nazareno Paolocci, George W. Booz

**Affiliations:** 1 Departments of Pharmacology and Toxicology, School of Medicine, and Center for Excellence in Cardiovascular-Renal Research, The University of Mississippi Medical Center, Jackson, Mississippi, United States of America; 2 Department of Chemistry and Biochemistry, Faculty of Sciences, Lebanese University, Rafic Hariri Educational Campus, Hadath, Lebanon; 3 Division of Cardiology, Johns Hopkins Medical Institutions, Baltimore, Maryland, United States of America; 4 Department of Medicine, Division of Cardiology, Centre Hospitalier Universitaire de Nancy, Brabois, France; 5 Department of Biochemistry, School of Medicine, and Center for Excellence in Cardiovascular-Renal Research, The University of Mississippi Medical Center, Jackson, Mississippi, United States of America; 6 Department of Chemistry, Wake Forest University, Winston-Salem, North Carolina, United States of America; 7 Clinical Medicine Department, Section of General Pathology, University of Perugia, Perugia, Italy; Leiden University Medical Center, The Netherlands

## Abstract

We previously showed that oxidative stress inhibits leukemia inhibitory factor (LIF) signaling by targeting JAK1, and the catalytic domains of JAK 1 and 2 have a cysteine-based redox switch. Thus, we postulated that the NO sibling and thiophylic compound, nitroxyl (HNO), would inhibit LIF-induced JAK-STAT3 activation. Pretreatment of human microvascular endothelial cells (HMEC-1) or neonatal rat cardiomyocytes with the HNO donors Angeli’s salt or nitrosocyclohexyl acetate (NCA) inhibited LIF-induced STAT3 activation. NCA pretreatment also blocked the induction of downstream inflammatory genes (e.g. intercellular adhesion molecule 1, CCAAT/enhancer binding protein delta). The related 1-nitrosocyclohexyl pivalate (NCP; not a nitroxyl donor) was equally effective in inhibiting STAT3 activation, suggesting that these compounds act as thiolate targeting electrophiles. The JAK1 redox switch is likely not a target of acyloxy nitroso compounds, as NCA had no effect on JAK1 catalytic activity and only modestly affected JAK1-induced phosphorylation of the LIF receptor. However, pretreatment of recombinant human STAT3 with NCA or NCP reduced labeling of free sulfhydryl residues. We show that NCP in the presence of diamide enhanced STAT3 glutathionylation and dimerization in adult mouse cardiac myocytes and altered STAT3 under non-reducing conditions. Finally, we show that monomeric STAT3 levels are decreased in the Gαq model of heart failure in a redox-sensitive manner. Altogether, our evidence indicates that STAT3 has redox-sensitive cysteines that regulate its activation and are targeted by HNO donors and acyloxy nitroso compounds. These findings raise the possibility of new therapeutic strategies to target STAT3 signaling via a redox-dependent manner, particularly in the context of cardiac and non-cardiac diseases with prominent pro-inflammatory signaling.

## Introduction

Cytokines that signal through the shared receptor gp130 play a critical role in promoting or suppressing inflammation. As such, these cytokines are involved in cancer, the immune response, wound healing, and ischemia or ischemia/reperfusion (IR) injury [1]. gp130 cytokines include interleukin-6 (IL-6) and leukemia inhibitory factor (LIF), which signal through homodimers of gp130 and heterodimers of gp130 with the LIF receptor (LIFR), respectively [2]. A notable feature of these cytokines is their prominent activation of the transcription factor signal transducer and activator of transcription 3 (STAT3). The Janus kinase JAK1, which is pre-associated with gp130 and the LIFR, is activated by transautophosphorylation as a result of dimer formation. Activated JAK1 phosphorylates STAT3 binding sites on gp130 and LIFR. Once recruited to those sites, STAT3 is phosphorylated by JAK1 on Y705, leading to its dimerization, translocation to the nucleus, and increased transcription. In the heart, STAT3 activation has been implicated in protection of the myocardium by ischemic and pharmacological pre- and post-conditioning [3]–[6]. In coronary endothelial cells and microvascular endothelial cells in general, STAT3 has been linked to protection of cardiac myocytes from IR injury and regulation of the inflammatory response [7]–[11].

Although JAK-STAT3 signaling in the heart is involved in cardiac protection and proper control of the immune and inflammatory responses, we previously found that this signaling pathway is inhibited by chronic oxidative stress, specifically by reactive oxygen species (ROS) formation [12]. Treatment of cardiac myocytes with parthenolide induced ROS formation and inhibited JAK1 activation by IL-6 and LIF. Recently, mutational analysis of the JH1 catalytic domain of the JAKs identified two closely positioned cysteine residues that form a redox-sensitive switch that is targeted by oxidative stress [13]. These observations raise the possibility that the beneficial actions of gp130 signaling in cardiac myocytes may be compromised under conditions associated with increased ROS formation, such as pathological cardiac hypertrophy and heart failure [14]. The role of STAT3 in vascular endothelial cells of the heart is less well defined. There are some reports that in these cells STAT3 activation is linked to an inflammatory response [15], but more reports that endothelial cellsTAT3 in the heart is linked to a protective and pro-angiogenic response at least in the context of ischemia/reperfusion injury [16]–[18].

Recent evidence has been reported that nitroxyl (HNO) donors may have therapeutic benefit in heart failure. In animal models, this one-electron reduction product of nitric oxide has been found to have positive inotropic/lusitropic effects under normal and congestive heart failure conditions [19]–[21]. The distinguishing characteristic of HNO is its “thiophylic" nature, allowing it to interact with redox-sensitive protein cysteines with high specificity [21], [22]. Based on new evidence for the occurrence of a cysteine-based redox switch in JAK1, we explored the possibility that HNO donors would modulate gp130 cytokine signaling. Here we report evidence that LIF-induced JAK-STAT signaling is inhibited by HNO donors, but STAT3 and not JAK1 is the primary target.

**Figure 1:**
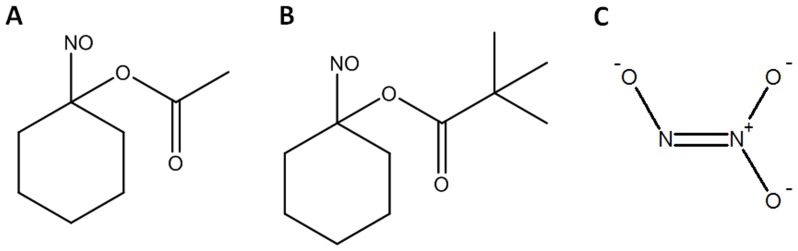
The acyloxy nitroso compounds, NCA and NCP, and the prototypical HNO donor, Angel’s salt, target STAT3. Chemical structures of (A) 1-Nitrosocyclohexyl acetate, NCA, (B) 1-nitrosocyclohexyl pivalate, NCP, and (C) Angeli’s salt (AS).

**Figure 2:**
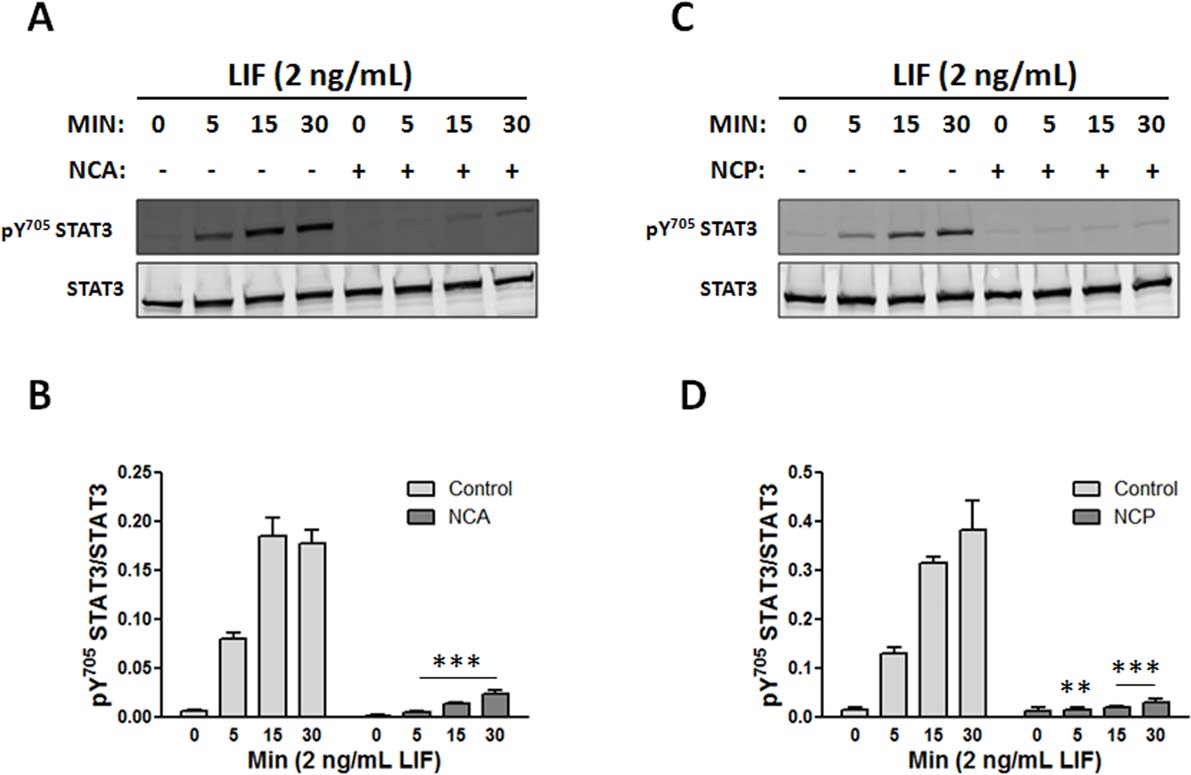
NCA and NCP inhibit LIF-induced STAT3 activation in human microvascular endothelial cells. HMEC-1 were pretreated for 1 h with vehicle (0.04% v/v DMSO), (A) 100 µM NCP, or (C) 100 µM NCP. Afterwards, cells were dosed for various times with 2 ng/mL LIF. Western immunoblots of cell lysates were probed for STAT3 Y705 phosphorylation and STAT3 as a loading control. (B and D) Results were quantified and expressed as the ratio of phosphorylated STAT3 to total STAT3. **P<0.01 and ***P<0.001 vs. same time point control (n = 4); 2-way ANOVA and Bonferroni post-test.

**Figure 3:**
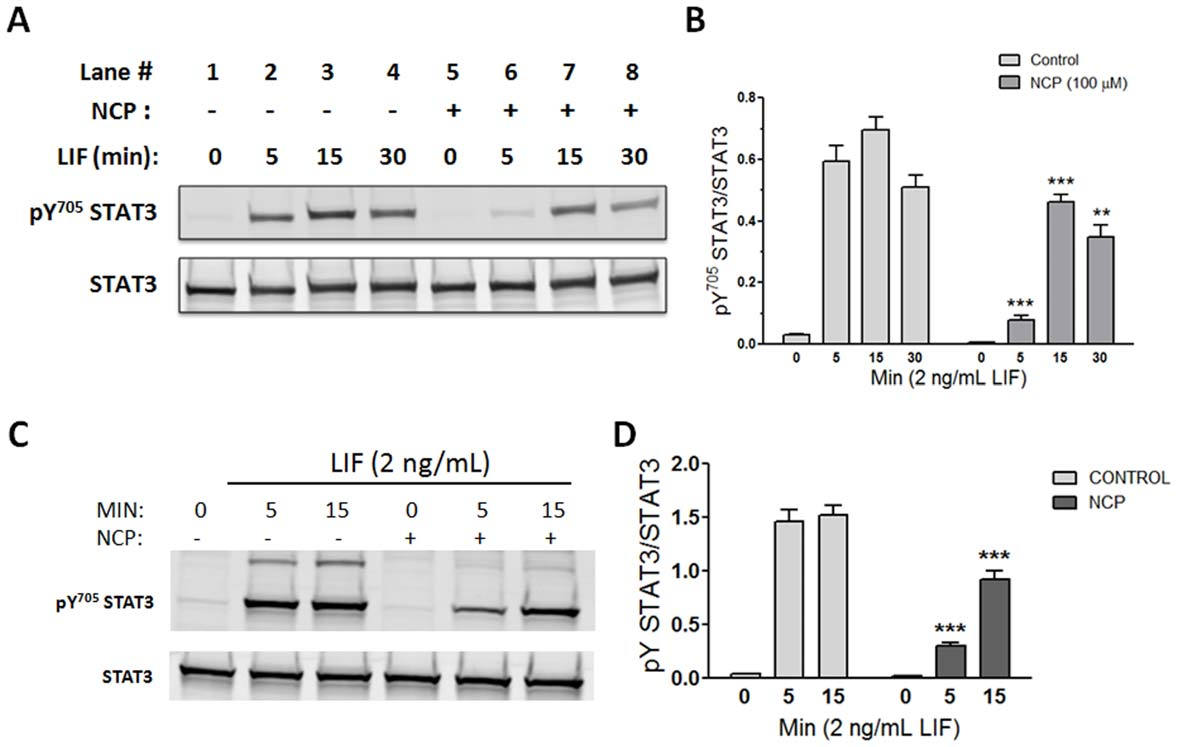
NCP inhibits LIF-induced STAT3 activation in cardiac myocytes. Neonatal rat ventricular myocytes (A & B) were pretreated for 1 h with 100 µM NCP (lanes 5–8) or vehicle (0.04% v/v DMSO; lanes 1–4). Cells were then dosed with 2 ng/mL LIF for various times. Western immunoblots of cell lysates were probed for STAT3 Y705 phosphorylation and STAT3. (A) Representative immunoblot of 4 independent experiments. (B) Compiled data analysis. Adult mouse cardiac myocytes (C & D) were pretreated for 1 h with 500 µM NCP (+) or vehicle (−). Cells were then dosed with 2 ng/mL LIF for 0, 5, or 15 min. (C) Representative immunoblot of 3 independent experiments. (D) Compiled data analysis. **P<0.01 or ***P<0.001 vs. same time point control; 2-way ANOVA and Bonferroni post-test (n = 3).

## Methods

### Materials

Cell culture reagents and alamarBlue were from Invitrogen (Carlsbad, CA, USA). Antibodies for total STAT3 (Cat. #9139), pY705 STAT3 (Cat. #9131), and phospho-p44/42 MAPK (ERK1/2) (Cat. #9106) were from Cellsignaling Technology (Danvers, MA, USA). 4G10 platinum anti-physphotyrosine monoclonal antibody, anti-cysteine-sulfenic acid antibody, and human recombinant LIF were from Millipore (Billerica, MA, USA). Antibodies to the LIF receptor (LIFR; Cat. #sc-659) and ERK1 (Cat. #sc-94) were from Santa Cruz Biotechnology (Santa Cruz, CA. USA), as were normal rabbit IgG and protein A/G PLUS agarose. Dimethyl sulphoxide (DMSO) Hybri-Max, hydrocortisone, dimedone, sodium *ortho*-iodosobenzoate (*o*-IBZ), and protease inhibitor cocktail (Cat. #P8340) were from Sigma-Aldrich (St. Louis, MO, USA). Angeli’s salt (AS) was a generous gift of Dr. Jon M. Fukuto (Sonoma State Univ.). Fluorescein-5-maleimide was from Pierce Biotechnology (Rockford, lL USA) and recombinant human STAT3 was from OriGene Technologies (Rockville, MD). Kinase extraction buffer (Cat. #BP-116K) and activated vanadate were purchased from Boston BioProducts (Ashland, MA, USA). Epidermal growth factor (EGF) was from BD Biosciences (San Jose, CA, USA). 1-Nitrosocyclohexyl acetate (NCA) and 1-nitrosocyclohexyl pivalate (NCP) were synthesized as described [23].

**Figure 4:**
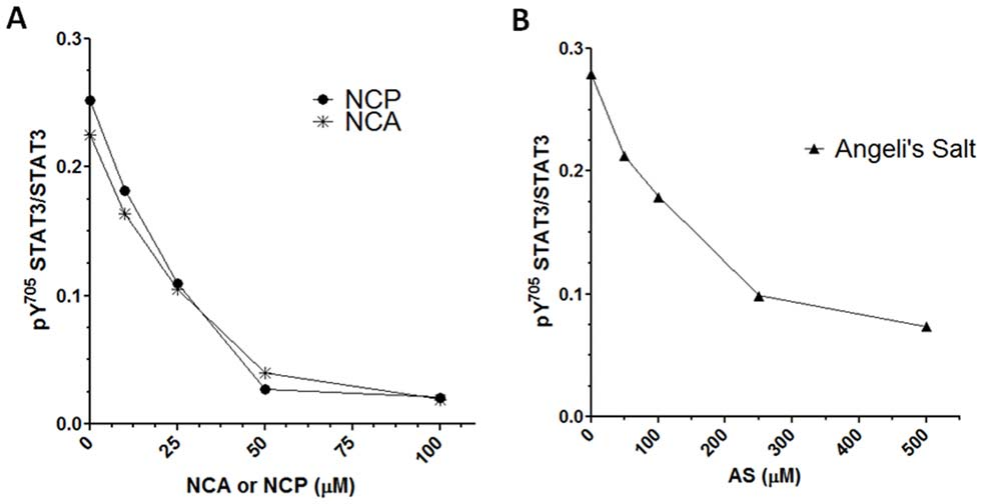
Dose response curves for inhibition of LIF-induced STAT3 activation by NCA, NCP and Angeli’s salt (AS). HMEC-1 were pretreated (A) for 1 h with various doses (0−100 µM) of NCA or NCP and the same amount of vehicle (0.04% v/v DMSO) or (B) for 30 min with 0−500 µM Angeli’s salt and the same amount of vehicle (50 µM NaOH). Cells were treated for 15 min with 2 ng/mL LIF. Western immunoblots of cell lysates were probed for STAT3 Y705 phosphorylation and STAT3. Results represent 2 independent experiments for both NCA and NCP and a single experiment for Angeli’s salt.

### Experimental Protocol

Human microvascular endothelial cells (HMEC-1) were obtained from the Centers for Disease Control and Prevention (CDC) and grown in MCDB 131 supplemented with 15% fetal bovine serum (FBS), 10 ng/mL EGF, 10 mM glutamine, 1 µg/mL hydrocortisone, and antibiotic-antimycotic. For experiments, cells were grown on 60 or 100 mm dishes to near confluency and incubated in medium with 0.5% FBS for 12–15 hours beforehand. Ventricular myocytes were isolated from 1–2 day-old Sprague-Dawley rat pups and maintained as described [12]. The study protocol was approved by the Institutional Animal Care and Use Committee. Experiments were performed 3–4 days later on cells incubated overnight in serum-free medium. At this time, the cardiac myocytes have developed a mature muscle phenotype characterized by intercellular connections and spontaneous and synchronous beating. Neonatal rat ventricular myocytes are widely used when studying the response of cardiac myocytes to stresses of different etiologies or hypertrophic signaling [12]. Cells were pre-incubated with NCA, NCP, or vehicle (0.04% DMSO) for 1 h, and treated with 2 ng/mL LIF for times indicated. Cells were pretreated with AS or vehicle (50 µM NaOH) for 15 min. Immunoprecipitations and Western analyses on cell lysates were carried out as described [12], [24]. Cell viability was measured using alamarBlue and cell proliferation was determined by cell count using a NucleoCounter [25]. HL-1 cells [26] were cultured in Claycomb medium supplemented with 10% FBS, 0.1 mM norepinephrine and penicillin/streptomycin. Subconfluent cultures were starved in serum- and norepinephrine-free medium for 24 for being used in experiments.

**Figure 5:**
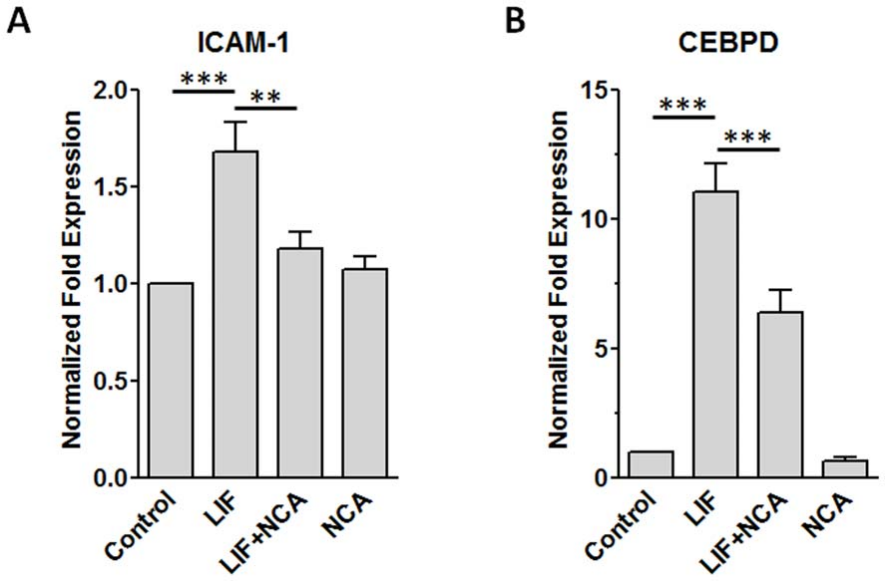
NCA attenuates LIF-induced gene expression. HMEC-1 were pretreated with vehicle (0.04% v/v DMSO) or 100 µM NCA for 1 h and then treated 1 h with 2 ng/mL LIF or vehicle. RNA was extracted, reverse transcribed, and analyzed by real time PCR for (A) ICAM-1 and (B) CEBPD expression. Results were normalized to GAPDH and expressed as the fold-increase over control levels. Values are mean ± SEM for (A) 7 or (B) 9 independent experiments. **P<0.01 and ***P<0.001, 1-way ANOVA and Newman–Keuls post-test.

**Figure 6:**
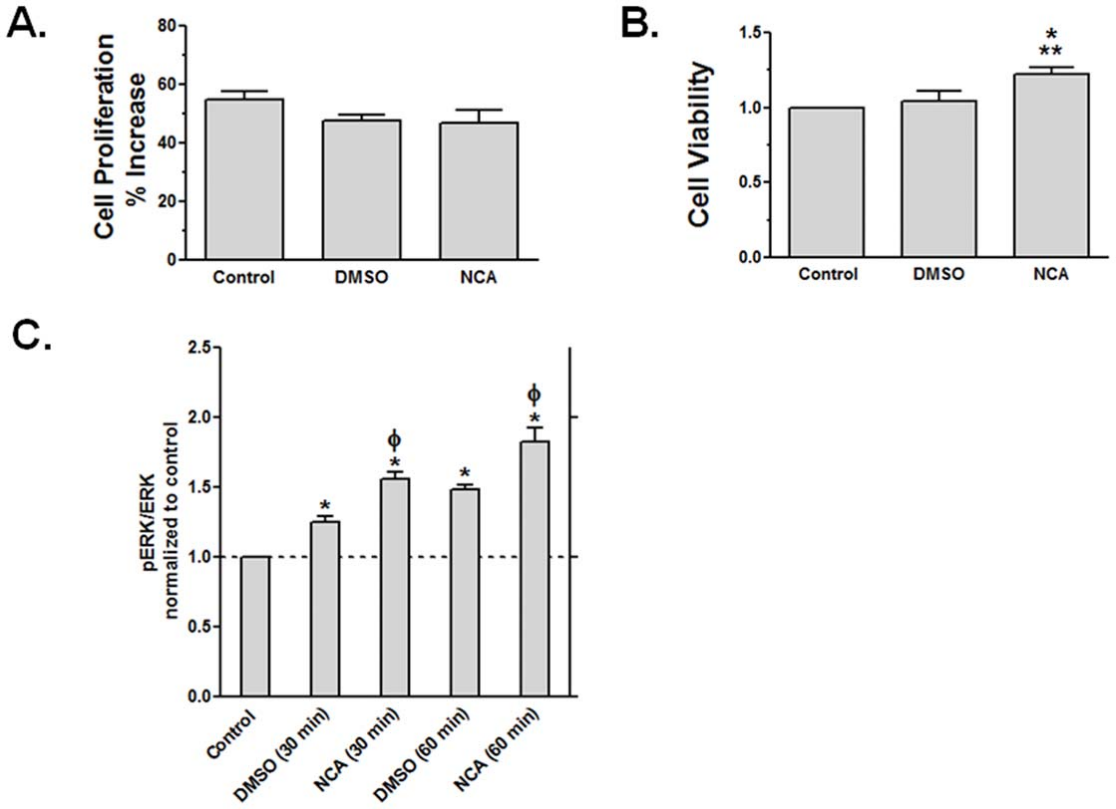
Effect on NCA on cell proliferation and viability. HMEC-1 cells were treated for 24 h with nothing (Control), vehicle (0.04% v/v DMSO), or 100 µM NCA. (A) Cell proliferation was assessed as the increase in live cells. (B) Cell viability was assessed by alarmarBlue assay. Results were normalized to control values for alamarBlue reduction. Values are mean ± SEM for (A) 4 and (B) 5 independent experiments, each performed using triplicate dishes of cells per condition. *P<0.05 and **P<0.01 vs. DMSO and Control, respectively; 1-way ANOVA and Newman–Keuls post-test. (C) NCA increased ERK1/2 phosphorylation as assessed by Western analysis. HMEC-1 were not treated (Control) or treated for 30 or 60 min with vehicle (0.04% v/v DMSO) or 100 µM NCA. Values are mean ± SEM for 4 independent experiments. *P<0.01 vs. Control and ^Φ^P<0.01 vs. timed vehicle (DMSO); 1-way ANOVA and Newman–Keuls post-test.

### Western Blots

Whole-cell lysates were prepared by scraping cells into ice-cold RIPA-based buffer containing 1 mM vanadate and protease inhibitor cocktail and were cleared by centrifugation at 100,000 g for 20 min at 4°C. Lysate samples containing equal amounts of proteins in Laemmli’s-SDS reducing buffer were separated by SDS-PAGE. For immunoprecipitations, whole cell extracts containing equal amounts of protein were incubated on a rotator with LIFR antibody for 3 h at 4°C. Protein A/G PLUS agarose was added and incubation continued overnight at 4°C. Agarose beads were washed 2x with ice-cold phosphate buffered saline and resuspended for SDS-PAGE in Laemmli’s reducing buffer. Separated proteins were blotted onto nitrocellulose membranes and the immunoreactive bands quantified using the Li-COR Odyssey infrared imaging system.

**Figure 7:**
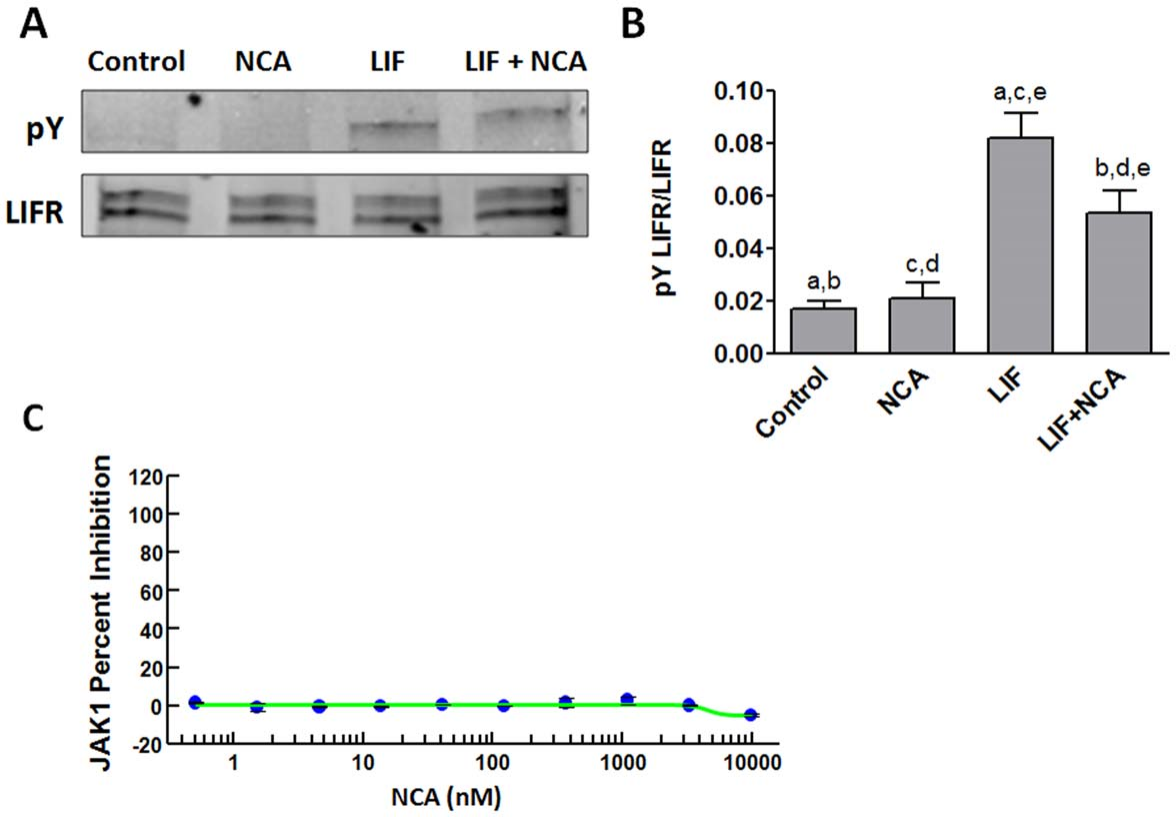
Effect of NCA on JAK1 activity. (A) LIFR phosphorylation was modestly decreased by NCA. HMEC-1 cells were pretreated with 100 µM NCA for 1 h followed by treatment with 2 ng/mL LIF for 10 min. Proteins were extracted and LIF receptor immnoprecipitated. Levels of phosphorylated tyrosine (pY) and LIFR were evaluated by Western blotting. (B) Quantification of the immunoblots. Values are mean ± SEM for 3 independent experiments. Columns with the same letter are significantly different from each other. ^b,d,e^P<0.05 and ^a,c^P<0.001; 1-way ANOVA and Newman–Keuls post-test. (C) NCA did not affect JAK1 catalytic activity. A FRET-based Z′-LYTE Assay (Invitrogen SelectScreen Profiling Service) was performed to assess the effect of various concentrations of NAC (0.51 nM –10 µM) on the catalytic activity of JAK1.

### Effect of NCP on Western Blot Analysis of Cardiac STAT3

The ventricles was harvested from the heart of a 2–3 month old male C57BL/6 mice (∼27 g) and homogenized in 1.2 mL TSE buffer (20 mM Tris, pH 7.4, 250 mM sucrose, and 1 mM EDTA) with protease inhibitors and vanadate. Debris was removed by centrifugation at 17,000 g for 15 min at 4°C. Four 200 µL aliquots of the supernatant were incubated in the dark with vehicle, 500 µM NCP, 1 mM diamide, or 500 µM NCP+1 mM diamide for 30 min at 22°C with constant rotation. The reaction was stopped by adding 400 µL ice-cold TSE buffer. A 75 µL aliquot was added to 25 µL 4x nonreducing Laemmli’s sample buffer and another aliquot to 25 µL 4x reducing Laemmli’s sample buffer with 2-mercaptoethanol. Both sets were boiled at 102°C for 5 min to disrupt noncovalent bonds. Proteins were separated by SDS-PAGE and transferred to nitrocellulose membranes, which were immunoblotted for STAT3 using a mouse monoclonal antibody from Santa Cruz (#sc-8019) raised against amino acids 50–240 in the N-terminus of STAT3. Detection was by the Li-COR Odyssey detection system.

### Fluorescein Labeling and JAK1 Kinase Activity

Human STAT3 (0.85 ng/µL) was treated with vehicle (DMSO), NCA (100 µM), or NCP (100 µM) for 1 h at room temperature in neutral redox buffer (1% Triton X-100; 50 mM NaCl; 10 mM Hepes; pH 7.4) and then labeled with 180 µM fluorescein-5-maleimide for 2 h in the dark. Afterwards, equal amounts of protein in Laemmli’s-SDS reducing buffer were separated by SDS-PAGE and fluorescence in the gel detected at 518 nm using a Biorad Chem Doc XRS Universal Hood. To ensure that samples contained equal amounts of proteins and to rule out non-specific effects, Western blot analysis was done on each fluorescein-labeled sample as well. Separated proteins on nitrocellulose membranes were blotted with a STAT3 antibody and imunoreactive bands quantified using the Li-COR Odyssey infrared imaging system. The effect of various concentrations of NCA (0.51 nM –10 µM) on the catalytic activity of JAK1 was performed by Invitrogen’s SelectScreen Profiling Service using the FRET-based Z′-LYTE Assay with an ATP concentration of K_m,app_.

**Figure 8:**
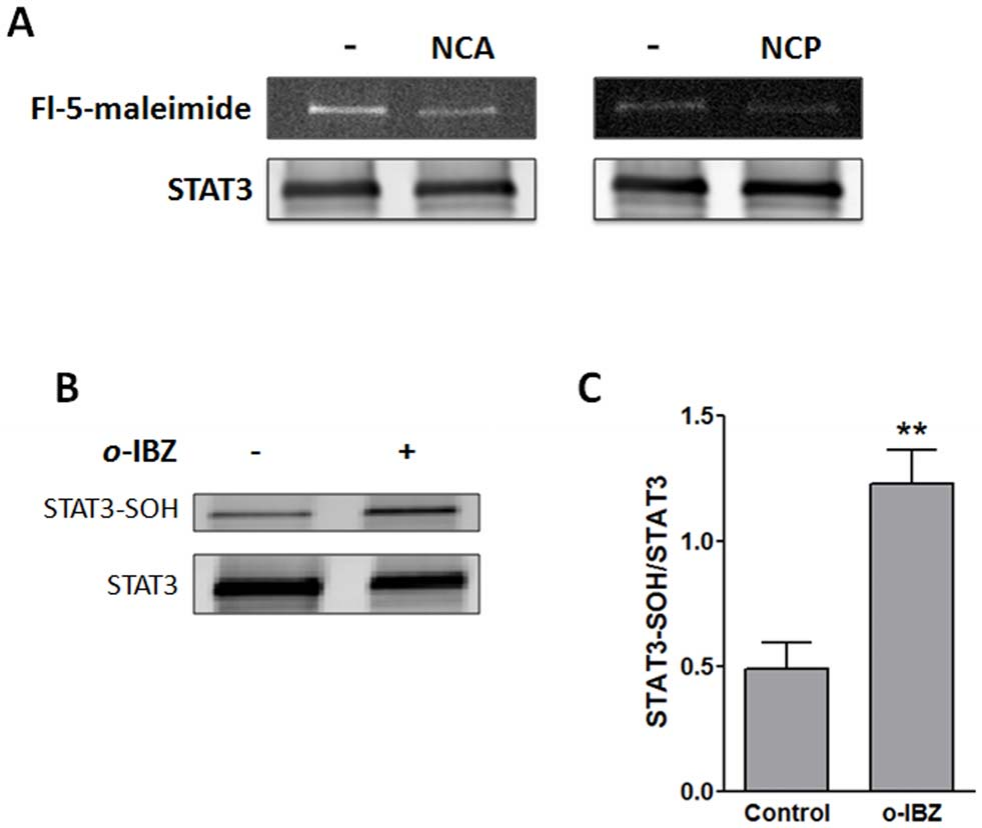
STAT3 possesses redox-sensitive cysteines. (A) NCA and NCP block thiolate labeling. Recombinant human STAT3 was treated with vehicle (DMSO), NCA (100 µM) or NCP (100 µM) for 1 h at room temperature and then labeled for 2 h with fluorescein-5-maleimide. Equal amounts of protein were separated by SDS-PAGE and fluorescence in the gel detected (upper panel). To ensure equal loading, Western analysis was done on each fluorescein-labeled sample. Separated proteins on nitrocellulose membranes were probed with a STAT3 antibody and imunoreactive bands quantified using the Li-COR Odyssey infrared imaging system (lower panel). Results shown are representative of 3 independent experiments. (B & C) Oxidation of STAT3 is associated with sulfenic acid formation. Purified recombinant STAT3 was immunoprecipitated and pretreated with 10 mM DTT and then treated with nothing or the oxidant *o*-IBZ (2.5 mM) for 1 hr at 4°C. Immunoprecipitates were processed as described under “Materials and Methods" to determine sulfenic acid formation (STAT3-SOH). (B) Representative blot. (C) Levels of cysteine-sulfenic acid and STAT3 were quantified by the Li-COR Odyssey Detection System. Treatment with *o*-IBZ resulted in a significant increase in relative sulfenic acid content. **P<0.01 vs. control, n = 3; paired Student’s t-test.

### STAT3 Protein-sulfenic Acid Detection

Sulfenic acid formation in STAT3 was detected as described [27]. Briefly, 100 ng of recombinant human STAT3 in RIPA-based extraction buffer with vanadate and protease inhibitors was incubated at 4°C under constant rotation with anti-STAT3 antibody. Protein A/G PLUS-agarose beads were added after 4 h and the incubation continued for 16 h. Immunoprecipitates were collected by centrifugation at 3000 g for 5 min and washed 3x for 5 min at 4°C with redox neutral wash buffer (1% Triton X-100, 0.1 mM sodium vanadate, 50 mM NaCl, 10 mM HEPES, pH 7.4). Immunoprecipitates were resuspended in wash buffer and split into 2 equal portions, which were treated with 10 mM DTT for 1 hour at 4°C under rotation. Complexes were washed 3x with a 5,000 g for 5 min centrifuge spin between washes and resuspended in wash buffer. Redox neutral wash buffer was added to one tube and 5 mM *o*-IBZ to the other. Samples were incubated for 1 hour at 4°C with rotation. Afterwards, complexes were washed 3x, and then incubated for 2 h with 10 mM dimedone at 4°C. The recovered immunoprecipitation complexes were resolved by SDS-PAGE and transferred to Li-COR nitrocellulose membranes. The ratio of sulfenic acid to STAT3 was determined with the Li-COR Odyssey Detection System using anti-STAT3 and anti-cysteine-sulfenic acid antibodies.

**Figure 9:**
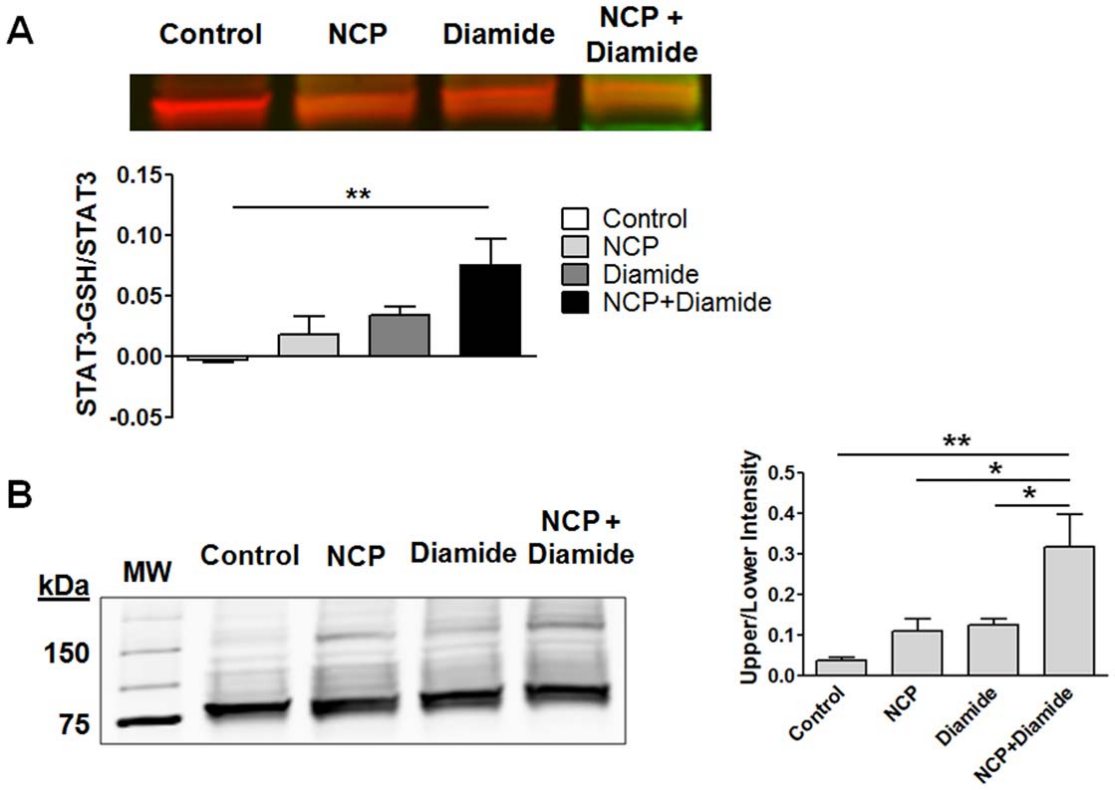
NCP enhances STAT3 glutathionylation and dimerization. HL-1 cells were treated for 30 min with vehicle (control), 500 µM NCP, 1 mM diamide, or 500 µM NCP and 1 mM diamide together. Cell extracts were prepared. (A) Equal protein amounts of cleared extracts were added to non-reducing Laemmli’s SDS-sample buffer and subjected to SDS-PAGE. Blots were probed for total STAT3 and glutathionylated protein using a rabbit and mouse antibody, respectively. Immunoreactive bands were detected using Li-COR Odyssey system and secondary antibodies that produced a red (anti-rabbit) or green (anti-mouse) signal. The overlay of the red and green signals produced an orange color. Relative levels of glutathionylated STAT3 were quantified. **P<0.01, 1-way ANOVA and Dunnett’s multiple comparison test (n = 3). (B) Cells were treated as in panel A. Cell extracts were added to non-reducing Laemmli’s SDS-sample buffer and subjected to SDS-PAGE. Blots were probed for total STAT3, which showed two bands consistent with STAT3 monomers and dimers. The intensity of the higher (dimer) band relative to the lower (monomer) band for each lane was quantified. *P<0.05 and **P<0.01, 1-way ANOVA and Newman–Keuls post-test (n = 3).

### Real Time PCR

RNA was extracted using the Ambion RNAqueous kit from Life Technologies (Carlsbad, CA) and cDNA prepared using the SuperScript VILO cDNA synthesis kit from Invitrogen. Gene amplification was carried out with the Applied Biosystems’ TaqMan Gene Expression Master Mix and gene expression measured using TaqMan gene expression assays for intercellular adhesion molecule 1 (ICAM-1, Hs_00164932_m1) and CCAAT/enhancer binding protein (C/EBP), delta (CEBPD, Hs_00270931_s1) from Life Technologies. Real time PCR was carried out with the Bio-Rad iQ5. Gene expression was normalized using the housekeeping gene GAPDH (Hs99999905_m1) from Applied Biosystems. Data were analysed with iQTM 5 optical system software from Bio-Rad (Hercules, CA).

### Assessment of STAT3 Redox Profile in a Model of Heart Failure

Transgenic mice over-expressing the Gαq subunit (Gαq) were used as a model of cardiomyopathy with systolic dysfunction [28]. Cardiac tissue from Gαq and non-transgenic (FVB/N background) littermates (n = 3; 4 months old) was extracted into 315 mM sucrose, 10 mM Tris (pH 7.5), 1 mM EDTA using a polytron homogenizer and centrifugated at 17,000×g for 10 minutes. Supernatants were obtained and protein determined using the BCA protein assay (Pierce). Samples were diluted into Laemmli sample buffer, split into fractions with and without β-mercaptoethanol (3.75% v/v) and boiled for 5 minutes. Extracts were run on Nupage 4–12% Bis_Tris (Life Technologies) gels using MOPS running buffer and western blots to STAT3 were performed using the Li-COR Odyssey scanner. Total protein loaded per lane was examined using the direct blue 71 staining process which has been previously reported to exhibit high sensitivity and a large linear dynamic range [29]. The relative protein loaded in each lane was determined by scanning membranes using a bed scanner (16 bit images) and quantitated using ImageJ software (NIH). The abundance of STAT3 in each lane was normalized to the total protein and the relative signal for STAT3 between non-reduced and reduced conditions was plotted.

**Figure 10:**
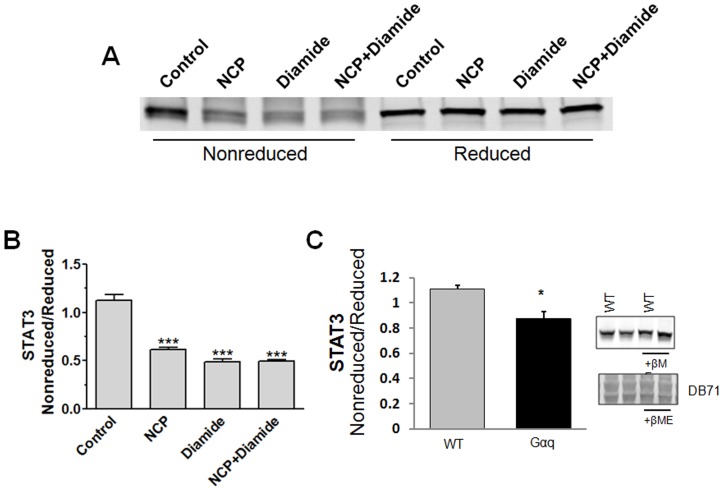
Oxidative stress, NCP and diamide alter the Western blot profile of STAT3 under nonreducing conditions. (A & B) Aliquots of a cleared mouse heart homogenate were incubated for 30 min with vehicle, 500 µM NCP, 1 mM diamide, or 500 µM NCP+1 mM diamide. Samples were processed for SDS-PAGE and Western blot analysis in nonreducing or reducing sample buffer. (A) Membranes were probed for STAT3 using the Li-COR Odyssey detection system. (B) Intensity of the STAT3 band in the nonreduced sample was normalized to the intensity of the band after reduction. ***P<0.001 vs. Control, 1-way ANOVA and Newman–Keuls post-test (n = 3 mouse hearts). (C) Ratio of nonreduced to reduced STAT3 in wild type (WT) and failing (Gaq) mouse hearts. STAT3 levels in mouse myocardial tissue from WT (FVB/N) and heart failure mice (Gaq overexpressing) (n = 3) were determined via immunoblot analysis under nonreducing or reducing (3.75% β-mercaptoethanol (β-ME)) conditions. Protein loads were normalized using the direct blue 71 stained membranes (DB71). *P<0.05 (Student t-test).

### Statistical Analysis

Results are expressed as mean ± SEM for n number of independent experiments or mouse hearts. Statistical significance involving multiple comparisons was assessed by one- or two-way ANOVA followed by an appropriate post-hoc test. The dimedone labelling and the effects of NCA and NCP on fluorescein labeling of JAK1 was evaluated using a paired t-test. A P value ≤0.05 was taken as significant.

## Results

### Acyloxy Nitroso Compounds Inhibit STAT3 Activation in Endothelial Cells and Cardiac Myocytes

The structures of the acyloxy nitroso compounds, NCA and NCP, and the prototypical HNO donor, AS, are shown in Figure 1. NCA is an HNO donor recently shown to hydrolyze to HNO under physiological conditions at a rate less than that of AS, while hydrolysis of NCP under similar conditions was essentially too slow to be significant [23]. However, like NCA and AS-derived HNO, NCP interacts with thiolates resulting in a number of cysteine oxidation modifications, such as disulfides or sulfinamide. HMEC-1 cells were pretreated for 1 h with vehicle (DMSO) or with either 100 µM NCA or NCP, and then treated with 2 ng/mL LIF for 0 to 30 min. STAT3 activity in cell extracts was determined by Western blot analysis. As Figures 2 shows, LIF induced a time-dependent increase in STAT3 activation as indexed by the fractional increase in STAT3 phosphorylated on Y705. Pretreatment with NCA or NCP was equally effective in attenuating LIF-induced STAT3 activation. NCP also inhibited LIF-induced STAT3 activation in cardiac myocytes (Fig. 3A), although in this case more residual activation was seen after pretreatment with 100 µM NCP than was observed in HMEC-1 (Fig. 3B). Differences in the potency of NCP between HMEC-1 and cardiac myocytes could be due to differences in the metabolism of this compound or levels of STAT3 expression. Finally, we observed that pretreatment of cardiac myocytes with 100 µM NCA decreased LIF-induced STAT3 Y705 phosphorylation at 5 min by 67.3±9.6% (n = 3, P<0.01). Notably NCP or NCA did not cause STAT3 S727 phosphorylation (data not shown), which we previously reported negatively affects subsequent STAT3 Y705 phosphorylation [30].

Using HL-1 cells, we assessed whether STAT3 activation was inhibited by NCP in adult cardiac myocytes as well. HL-1 is a mouse cardiomyocyte cell line that continuously divides and spontaneously contracts while maintaining a differentiated adult cardiac phenotype [31]. As seen from Figure 3C, pretreatment of HL-1 cells with NCP inhibited LIF-induced activation of STAT3. A higher concentration of NCP was required to see inhibition than was effective in HMEC-1 or neonatal rat ventricular myocytes, due perhaps to higher metabolism of NCP or a greater number of competing thiolate residues. In HL-1 cells, we observed that LIF-induced a higher molecular weight band that was reactive with the anti-pY705 STAT3 antibody and its induction was inhibited as well by NCP (Fig. 3C). The size of this higher band is consistent with that of a STAT3 dimer that would form upon STAT3 tyrosine phosphorylation or with a STAT3 pY705-Hsp90 complex, which we have observed is resistant to detergent disruption (unpublished observation).

### Dose-Response Curve for NCA, NCP, and AS

We next showed that LIF-induced Y705 STAT3 phosphorylation levels were decreased by NCA or NCP in a concentration-dependent manner (Fig. 4A). Both compounds exhibited similar potency in inhibiting STAT3 activation. We also showed that the prototypical nitroxyl donor, AS inhibited STAT3 phosphorylation in endothelial cells in a dose-dependent manner. In this case, cells were pretreated for only 30 min given the short life of AS in physiological medium [32]. AS/NO also inhibited STAT3 activation in endothelial cells, but at higher concentrations than required for NCA or NCP, likely due to the more labile nature of this compound (Fig. 4B).

### NCA Decreases LIF-Induced Gene Expression in Endothelial Cells

We next sought to establish whether NCA-induced attenuation of LIF-induced STAT3 activation impacted on the expression of pro-inflammatory genes linked to its activation. ICAM-1 is a membrane surface receptor important for the tissue infiltration of leukocytes and CEBPD is a transcription factor important in the activation of inflammatory genes. Cells were pretreated with NCA or vehicle and then treated with LIF or vehicle. RNA was extracted and the expression of ICAM-1 and CEBPD evaluated by real time PCR. Our results show that LIF increased expression levels of both ICAM-1 and CEBPD, and NCA pretreatment was effective in reducing this induction (Fig. 5). Next, we established that the effect of NCA on gene expression was not a result of a harmful effect on cell viability. We observed that prolonged treatment with NCA for 24 h had no adverse effects on the increase in cell number (Fig. 6A) and actually enhanced slightly cellular mitochondrial activity as assessed by alamarBlue reduction (Fig. 6B). By itself, NCA induced a modest increase in ERK1 and ERK2 phosphorylation (Fig. 6C), indicating that this compound was not an overall inhibitor of intracellular signaling.

### NCA Decreases Phosphorylation of the LIF Receptor

We previously reported that oxidative stress inhibits JAK1 tyrosine kinase activity [12]. Moreover, the JH1 catalytic domains of both JAK1 and JAK2 contain 2 conserved cysteine residues, which were recently shown through mutational analysis to function as a redox switch [13]. Thus, we asked whether the acyloxy nitroso compounds, which target redox-sensitive cysteine residues, were inhibiting STAT3 activation by targeting JAK1, which lies immediately upstream of STAT3 in LIF signaling. We first assessed the effect of NCA on LIF-induced phosphorylation of LIFR as a marker for its actions on JAK1, since JAK1 phosphorylates this receptor upon ligand binding to create sites for STAT3 recruitment. Cells were pretreated with NCA for 1 h followed by treatment with LIF for 10 min. Proteins were extracted and immunoprecipitation of LIFR performed. NCA decreased the tyrosine phosphorylation of LIFR observed in response to LIF stimulation of HMEC-1(Fig. 7A); however, the effect was not complete and significant phosphorylation of LIFR upon LIF stimulation was still seen after NCA treatment (Fig. 7B). We also found that NCA over a broad range of concentrations was without effect on the catalytic activity of the JH1 domain of JAK1 using a Z′-LYTE assay (Fig. 7C). Together these findings indicate that any inhibition of JAK1 by the acyloxy nitroso compounds does not result from a direct effect on the JH1 catalytic domain and does not adequately explain the inhibition of LIF-induced STAT3 tyrosine phosphorylation.

### NCA and NCP Inhibit Fluorescein-5-Maleimide Labeling of Recombinant STAT3

STAT3 was recently reported to be redox-sensitive [33], [34]. Since the actions of NCA on JAK1 activity were modest, we shifted our attention to STAT3 and asked whether NCA and NCP were directly targeting reactive thiolate residues in this transcription factor. Pretreatment with either NCA or NCP effectively reduced labeling of free sulfhydryl residues in recombinant human STAT3 with fluorescein-5-maleimide at physiological pH (Fig. 8). NCA reduced labeling by 18.5±0.5% (n = 3; P<0.01), and NCP by 14.2±1.6% (n = 3; P<0.01). To further establish that STAT3 is redox-sensitive, we asked whether the cysteine residues of STAT3 could form sulfenic acid, which occurs as an intermediate stage in the oxidation of cysteine in the catalytic center of enzymes and as a sensor of nitrosative and oxidative stress in some transcriptional regulators, transcription factors, and signaling proteins or kinases [35]. For these experiments, recombinant human STAT3 was first fully reduced with DTT. Treatment of reduced STAT3 with the oxidant *o*-IBZ was associated with enhanced labeling with dimedone, which specifically traps sulfenic acid (Fig. 8B). Oxidation of STAT3 resulted in a 161±33% (n = 3; P<0.01) increase in labeling of STAT3 for sulfenic acid (Fig. 8C).

### NCP Enhances STAT3 Glutathionylation and Dimerization

Protein glutathionylation protects cysteine thiolates and sulfenic acid residues from irreversible oxidation. Protein sulfenic acids are known to react with oxidized glutathione (glutathione disulphide) or reduced glutathione resulting in protein glutathionylation [36], [37]. Alternatively, protein gluathionylation can occur via the partial oxidation of the protein thiol or the thiol of glutathione to a thyil radical, which then can interact with glutathione or the protein thiolate, respectively [36]. If NCP were targeting thiolates of STAT3 for oxidation, we hypothesized that NCP would enhance STAT3 glutathionylation under conditions favoring glutathione oxidation to either glutathione disulphide or the thiolate radical, such as seen with diamide [38]. As seen from Figure 9A, that was in fact the case. Treatment of HL-1 cells with either NCP or diamide alone for 30 min did not cause significant STAT3 glutathionylation; however, when present together STAT3 glutathionylation was observed. Increased glutathionylation was associated as well with increased formation of a higher molecular weight band that was the size of a STAT3 dimer and may have resulted from intermolecular STAT3 disulfide bond formation. Thus NCP may protect STAT3 from further more irreversible oxidation.

### NCP Alters the Western Blot Profile of STAT3 under Nonreducing Conditions

We reasoned that if the acyloxy nitroso compounds were interacting with STAT3 via redox-sensitive cysteine residues, NCP would affect the detection of STAT3 by Western immunoblotting under nonreducing conditions. Homogenates of ventricles from mouse heart were prepared in a detergent-free buffer favoring preservation of protein-protein interactions and native protein conformations. Homogenates were treated with vehicle, NCP, diamide, or NCP and diamide for 30 min and then processed for Western analysis under both nonreducing and reducing conditions. No difference was seen among the treatments in the level of STAT3 detected under reducing conditions (Fig. 10A). However, NCP and diamide decreased levels of STAT3 detected in nonreduced samples (Fig. 10 A & B). Statistically, NCP was as effective as diamide in decreasing nonreduced STAT3 levels and no greater effect was seen with their combination.

Finally, we assessed whether oxidative stress associated with a pathological disease state impacted the redox profile of STAT3. As Figure 10C demonstrates, levels of detectable monomeric STAT3 assessed under nonreducing conditions were significantly decreased in the Gαq model of heart failure in a redox-sensitive manner. This observation is consistent with STAT3 forming redox-sensitive higher order complexes under conditions of increased oxidative stress and supports the conclusion that the redox-sensitivity of STAT3 has pathophysiological significance.

## Discussion

Here we report that two newly-described acyloxy nitroso compounds that target reactive cysteine residues inhibit LIF-induced STAT3 activation. We provide evidence that STAT3 is a direct target of these compounds, supporting the idea that STAT3 is redox-sensitive. In addition, we show for the first time that STAT3 can form sulfenic acid during its oxidation, which is a feature of redox-sensitive proteins. Our study findings may have at least two major pathophysiological implications: First, STAT3 signaling in the heart may be modulated by redox status and attenuated by oxidative stress as in heart failure. This contention is supported by present evidence showing that hearts isolated from mice with cardiac selective over-expression of Gαq protein [28] display lower levels of monomeric STAT3 under non-reducing conditions, in agreement with the high oxidizing conditions found in these mice [39]. Second, when given acutely nitroxyl donors may attenuate or inhibit LIF-induced STAT3 activation that is a necessary for inducing the gene expression of pro-inflammatory molecules such as ICAM-1 and CEBPD. The latter finding suggests that HNO donors may benefit the heart in all diseased conditions characterized by accentuated inflammatory states.

The mechanism by which the acyloxy nitroso compounds inhibit STAT3 activation will require extensive investigation. In this regard, it is important to point out however that the acyloxy nitroso compounds NCP and NCA do not give S-nitroso thiols (nitrosylation) [23]. The compounds may prevent STAT3 interaction with gp130 or LIFR or the phosphorylation of STAT3 by JAK1. Either of these possibilities could be due to the introduction of an oxidized cysteine modification in STAT3, such as a sulfinic acid or disulfide. Consistent with that possibility is the report of others that *S*-glutathionylation of STAT3 *in vitro* rendered it a poor substrate for recombinant JAK2 [40]. No evidence to date has been found to indicate that the acyloxy nitroso compounds form protein cysteine adducts (S. B. King, unpublished observation). Alternatively, the oxidative actions of these compounds on STAT3 cysteine residues could lead to intra- or intermolecular disulphide bonds that would preclude STAT3 association with gp130 or LIFR. However, here we show that the acyloxy nitroso compound NCP alone did not cause significant STAT3 gluathionylation or dimer formation (Fig. 9).

Further evidence that STAT3 has redox-sensitive cysteine residues was obtained using STAT3 from mouse hearts. Pretreatment of tissue homogenates with the acyloxy nitroso compound NCP or the thiol oxidizing agent diamide reduced detection of STAT3 by Western analysis under nonreducing conditions. This effect was reversed by reducing the samples with 2-mercaptoethanol. This finding is consistent with NCP or diamide causing formation of higher order cysteine residue-based complexes. Our cardiac STAT3 extraction procedure of homogenization in detergent-free buffer was intended to preserve STAT3 higher order complex formations. It should be pointed out that we think it unlikely that STAT3 glutathionylation occurred under the conditions employed in this assay as levels of glutathione and oxidized glutathione would have been effectively reduced by dilution of the cellular contents in the homogenization buffer.

The acyloxy nitroso compounds and nitroxyl are electrophiles [23], and there are several reports of IL-6 cytokine signaling being inhibited by electrophilic compounds that possess an α,β-unsaturated carbonyl group. Pretreatment for 30 min with two naturally occurring terpenes, dehydrocostuslactone and costunolide inhibited IL-6-induced STAT3 tyrosine phosphorylation in the human THP-1 cell line and lead to S-glutathionylation of STAT3; however, these compounds also inhibited upstream JAK activity [41]. Pretreatment for 30 min with the α,β-unsaturated flavonoid chalcone was reported to inhibit IL-6-induced STAT3 tyrosine phosphorylation in bovine aortic endothelial cells (BAEC) and GSH depletion with BSO enhanced this effect [42]. Unfortunately, the effect of chalcone on JAK activity was not reported. In BAEC, pretreatment for 30 min with the arachidonic acid metabolite and electrophile, 15-deoxy- Δ^12,14^–prostaglandin J_2_ (15d-PGJ_2_) inhibited IL-6-induced STAT3 Y705 phosphorylation, as did acrolein [43]. At least in the case of 15d-PGJ_2_, no inhibition of JAK activity was found. This study thus provides more definitive evidence that STAT3 is targeted by electrophiles. Yet in mouse embryonic stem cells, 15d-PGJ_2_ inhibited LIF-induced JAK1 activation [44], highlighting the point that in cells either JAK or STAT3 activation appear to be targeted by electrophilic compounds. In our study, we did not observe an effect of NCA on JAK1 catalytic activity and found a modest inhibition by NCA of LIFR tyrosine phosphorylation, a measure of JAK1 activity. Moreover, our results on recombinant STAT3 show for the first time that the acyloxy nitroso compounds directly target reactive cysteine residues in this transcription factor.

To our knowledge there are three studies that have demonstrated that STAT3 has redox-sensitive cysteine residues that impact on its function. Treatment of human HepG2 hepatoma cells with thiol targeting agents (diamide, glutathione disulfide, and pyrrolidine dithiocarbamate) was reported to inhibit IL-6-induced STAT3 activation and increase the proclivity of STAT3 to undergo glutathionylation [40]. These agents also decreased nuclear accumulation of STAT3 and impaired expression of STAT3-target genes. Peroxide was reported to induce STAT3 homodimer formation in HEK293 cells and a cysteine residue in the amino terminus was identified as the reactive residue involved [34]. A subsequent study employing mutational analysis identified a cluster of redox-sensitive cysteine residues in the DNA binding domain and one within the C-terminus transcription activation domain [33]. In a cellsystem, these residues were found to be responsible for peroxide-induced formation of STAT3 multimers and decreased IL-6-induced STAT3 activation and STAT3-mediated reporter gene expression, as well as attenuation of IL-6-enhanced binding of STAT3 to consensus serum-inducible elements (SIE) found in the c-*fos* promoter. Intriguingly, peroxide did not affect STAT3 recruitment to the c-*myc* P2 promoter, suggesting that STAT3 oxidation may determine the gene expression profile linked to this transcription factor.

Our findings that STAT3 possesses redox-sensitive cysteine residues that are targeted by small molecular weight molecules raises the possibility that drugs could be designed to selectively activate or block the impact of oxidative stress on the function of STAT3. Obviously, more research needs to be done to define the redox-related role of STAT3, which to date would seem likely to involve two distinct actions, mitochondrial and nuclear. A number of studies have shown the importance of mitochondrial STAT3 in the proper functioning of the electron transport chain and suppression of mitochondrial ROS formation [45]. In addition, recent studies have identified a role for unphosphorylated STAT3 (U-STAT3) in the induction of genes that are distinct from those linked to canonical (phosphorylated) STAT3 activation and important in sustaining an inflammatory response [46]. It is likely that the redox-sensitive nature of STAT3 is important in both of these noncanonical actions of STAT3.

The activation of the JAKs or STAT3 has been implicated in many important pathophysiological conditions, especially in the cardiovascular system [2]. This ranges from early- and late-preconditioning to the control of cardiac cell growth, under normal and stressed conditions. In these settings, alterations in myocardial and vascular redox balance may be present, in concomitance with pro-inflammatory conditions. Thus, the finding that STAT3 signaling is redox-sensitive and that HNO donors may counter this activation may have far-reaching translational implications in all cardiac diseased conditions characterized by structural and functional disarrangement in which pro-inflammatory signaling pathways are prominently at play.
